# Socioeconomic Disparities in the Prevalence of Blepharoptosis in the South Korean Adult Population Based on a Nationwide Cross-Sectional Study

**DOI:** 10.1371/journal.pone.0145069

**Published:** 2016-01-04

**Authors:** Eun Young Rha, Kyungdo Han, Yongkyu Park, Gyeol Yoo

**Affiliations:** 1 Department of Plastic and Reconstructive Surgery, Incheon St. Mary’s hospital, College of Medicine, The Catholic University of Korea, Seoul, Korea; 2 Department of Biostatistics, College of Medicine, The Catholic University of Korea, Seoul, Korea; 3 Department of Plastic and Reconstructive Surgery, Yeouido St. Mary’s hospital, College of Medicine, The Catholic University of Korea, Seoul, Korea; Institute for Health & the Environment, UNITED STATES

## Abstract

**Purpose:**

We investigated the association between socioeconomic status (SES) and the prevalence of blepharoptosis in a representative South Korean population.

**Methods:**

This cross-sectional study was based on data obtained in the Korea National Health and Nutrition Examination Survey from 2010 to 2012. In total, 17,178 Korean adults (7,261 men and 9,917 women) aged 19 years or older were enrolled. Blepharoptosis was defined as a marginal reflex distance 1 (MDR 1) lower than 2 mm. Household income and education level were used as indicators of SES. Univariate and multiple logistic regression analyses were conducted to analyze the relationship between SES and the prevalence of blepharoptosis.

**Results:**

Household income was inversely associated with the prevalence of blepharoptosis in women [adjusted odds ratio (aOR) and corresponding 95% confidence interval (95% CI) was 1.894 (1.336, 2.685)], and educational level was inversely associated with blepharoptosis in both men and women [aORs and 95% CIs were 1.572 (1.113, 2.219) and 1.973 (1.153, 3.376), respectively]. After adjusting for household income and educational level, low SES was associated with a high prevalence of blepharoptosis in women only.

**Conclusions:**

Socioeconomic disparities in the prevalence of blepharoptosis were found among women. Indeed, future research using a prospective design to determine the causal relationship between SES and blepharoptosis may identify SES as a risk factor for this condition.

## Introduction

The normal anatomic position of the upper eyelid margin is 0.5–2 mm below the superior corneal limbus [1]. Blepharoptosis is defined as an abnormally low-lying upper eyelid margin on primary gaze that results in the eyelid covering the pupil [1]. Blepharoptosis leads to both esthetic and functional problems. It causes a tired and aged appearance, blurred vision, and increased tearing. Significant blepharoptosis sometimes forces patients to tilt their heads back into a chin-up position and lift the drooping eyelid with a finger or raise their eyebrows to see. Because of continuous activation of the forehead and scalp muscles, tension headaches and eyestrain can occur [1]. Although blepharoptosis may present within a broad range of people, its prevalence increased with age and was highest in the age group older than 70 years [2].

Blepharoptosis has been classified into different subtypes according to age at onset, etiology, severity, and levator function [3, 4]. Congenital ptosis develops from levator maldevelopment, and myogenic ptosis develops as the result of diseases, such as myasthenia gravis and chronic progressive ophthalmoplegia. Neurogenic ptosis is an apraxia of lid opening caused by blepharospasm, cerebral vascular accidents, and multiple sclerosis, or other illnesses. Aponeurotic ptosis results from the disinsertion or dehiscence of the levator aponeurosis from the anterior surface of the tarsus. Mechanical ptosis develops as a result of scarring or excessive weight or eyelid and orbital mass. Traumatic ptosis can develop from severe facial trauma to the orbit or periocular tissues [3, 4]. In other words, blepharoptosis is a multifactorial condition, resulting from interaction between an individual’s genetic background, illness status, and environment, including social risk factors. However, there have been few reports about the epidemiology of blepharoptosis.

We conducted this study to investigate the relationship between elements of socioeconomic status (SES) and the prevalence of blepharoptosis in the South Korean population based on data from the Korea National Health and Nutrition Examination Survey (KNHANES) conducted in 2010 and 2012.

## Materials and Methods

### Study participants

This study was based on the data acquired from the 2010–2012 Korea National Health and Nutritional Examination Survey (KNHANES) V conducted by the Korea Centers for Disease Control and Prevention (KCDC). The KNHANES is an ongoing, population-based, cross-sectional survey in South Korea. A complex, stratified, multistage, cluster sampling design with proportional allocation based on the National Census Registry was used for the selection of household units. The samples were weighted to represent the non-institutionalized civilian Korean population.

Individuals participated in health interviews, health examination surveys, and nutrition surveys conducted at their homes. A total of 25,534 participants completed this survey. Those younger than 19 years of age (n = 4,890) were excluded, and an additional 3,466 participants were excluded due to missing values for variables related to ophthalmic examination, educational level, and household income, yielding a final study population of 17,178 participants (7,261 men and 9,917 women). The Institutional Review Board of The Catholic University of Korea approved this study (SC15QISE0091).

### Demographic Variables

All participants were asked about their demographic characteristics, socioeconomic characteristics (including household income, educational level, occupation, type of employment, national basic livelihood security status, and job status) and medical history by trained interviewers. Self-report questionnaires were used to determine their smoking status, alcohol consumption, physical activity, and occupation. Smoking status was classified into current smokers, ex-smokers, and non-smokers. Subjects who were currently smoking and had smoked > 100 cigarettes in their lifetime were defined as current smokers. In terms of alcohol consumption, participants were classified as non-drinkers (≤ 1 g/day), moderate drinkers (1.0–29.9 g/day), or heavy drinkers (≥ 30.0 g/day) according to the frequency and amount of alcohol consumed. Total caloric intake and the proportions of energy from carbohydrates, protein, and fat were also estimated. Physical activity was categorized as regular exercise and non-regular exercise. Regular exercise was defined as exercising more than three times per week for more than 20 minutes at a time. Height, weight, and waist circumference (WC) were measured with standard procedures. Height was measured with an accuracy of 0.1 cm, and weight was measured to the nearest 0.1 kg. WC was measured at the midpoint of the lower margin of the twelfth rib and the iliac crest in the mid-axillary line at the end of expiration. Weight was measured to the closet 0.1 kg, and body mass index (BMI) was calculated as weight in kilograms divided by height in meters squared. Hypertension was defined by blood pressure ≥ 140/90 mmHg or by the current use of antihypertensive medicines. Diabetes was defined by fasting plasma glucose levels ≥ 126 mg/dl with diabetes treatment or diagnosis by a physician. Metabolic syndrome was defined according to the American Heart Association Heart Association/National Heart, Lung, and Blood Institute Scientific Statement (AHA/NHLBI) criteria for Asians [5]. Occupation was categorized into seven groups according to the major categorizations of the 6^th^ Korean Standard Classification of Occupations: ‘managers, professionals, and administrators;’ ‘clerks;’ ‘service and sales workers;’ ‘skilled agricultural, forestry, and fishery workers;’ ‘equipment or machine operators and craft and assembling workers;’ ‘elementary workers;’ and ‘unemployed.’

### Socioeconomic Status

Educational level and household income were used to assess SES. Level of education was categorized as elementary school or lower (≤ 6 years), middle school (7–9 years), high school (10–12 years), and university and higher (≥ 13 years) according to the number of years of schooling. Household income was divided into quartiles and categorized as low (quartile 1 or Q1), medium–low (Q2), medium–high (Q3), and high (Q4) according to the monthly household equivalent income. Monthly income was categorized according to the number of family members (monthly income/√number of family members) [6].

### Ophthalmologic Examination

Blepharoptosis was defined as a marginal reflex distance (MRD) 1 of < 2 mm in at least one eye. MRD 1 was defined as the distance from the pupillary light reflex point to the upper eyelid margin. Participants were asked to look straight ahead and relax, focusing on a distant target. The corneal light reflex was observed after a penlight was aimed at the participant’s eye to illuminate the cornea, and the distance between the cornea and the upper eyelid margin was measured in millimeters.

### Statistical Analyses

All analyses were performed using SAS version 9.3 (SAS Institute Inc., Cary, NC, USA). General characteristics according to the blepharoptosis are presented as means ± standard errors of the mean (SEMs) for continuous variables and as percentages and standard errors (SEs) for categorical variables. The SAS survey procedure reflected the complex sampling design and the sampling weights of KNHANES and provided nationally representative prevalence estimates. A Student’s t-test or a Rao–Scott chi-square test was used for comparisons among groups. Multiple logistic regression analyses were used to estimate the prevalence odds ratios (ORs) and 95% confidence intervals (CIs) for blepharoptosis, which were calculated for each SES-related factor. Factors such as age, gender, education, household income, and other demographic variables were included in the model. Model 1 was adjusted for age; Model 2 was adjusted for the variables in Model 1 plus BMI, smoking and drinking, physical activity and occupation; and Model 3 was adjusted for the variables in Model 2 plus diabetes mellitus, family history of eye disease, and energy and fat intake. A *P* value of < 0.05 was considered statistically significant.

## Results

Table 1 presents the general characteristics of subjects according to gender and presence of blepharoptosis. The mean ages of men and women with blepharoptosis were 55.4 ± 0.9 and 65.1 ± 0.8 years, respectively. In both genders, subjects with blepharoptosis were older and were more likely to have hypertension, diabetes, metabolic disease, cataracts, and a family history of eye diseases and to be covered by national basic livelihood security compared with subjects without blepharoptosis.

**Table 1:** General characteristics of study subjects according to the gender.

	Men (n = 7261)			Women (n = 9917)		
	No blepharoptosis	Any blepharoptosis	P-value	No blepharoptosis	Any blepharoptosis	P-value
N	6317	944		8836	1081	
Age (years)	43.1±0.3	55.4±0.9	<.0001	44.4±0.3	65.1±0.8	<.0001
Smoking status: current smokers, n (%)	40.8(0.9)	37(2.1)	0.0958	4.8(0.3)	4.2(0.9)	0.534
Alcohol consumption: heavy drinkers, n (%)	17.8(0.7)	18(1.9)	0.8892	2.1(0.2)	0.2(0.2)	<.0001
Physical activity: regular exerciser, n (%)	23.6(0.8)	21.1(2.2)	0.3029	17.1(0.6)	13.4(1.5)	0.0342
BMI (kg/m^2^)	24.1±0.1	24.3±0.1	0.0571	23.2±0.1	24.3±0.1	<.0001
WC (cm)	83.9±0.2	86.3±0.3	<.0001	77.6±0.2	82.8±0.4	<.0001
Dietary intake						
Total energy (kcal/day)	2476±20.8	2205.3±42.7	<.0001	1721.9±10.8	1495.5±25.1	<.0001
Fat (% of energy)	19.8±0.2	16.6±0.5	<.0001	18±0.1	12.5±0.4	<.0001
Hypertension	29.5 (0.9)	47.6 (2.3)	<.0001	20.8 (0.7)	55.4 (2.3)	<.0001
Diabetes mellitus (%)	8.5(0.5)	19(1.8)	<.0001	6.3(0.4)	20.7(1.6)	<.0001
Metabolic syndrome (%)	24.9(0.7)	39.9(2.2)	<.0001	23.5(0.6)	56(2)	<.0001
History of cataracts, n (&)	23.1(0.9)	54.2(2.6)	<.0001	22.5(0.9)	72.1(2.4)	<.0001
Family history of eye disease, n (%)	79.3(0.8)	84.1(1.8)	0.0227	76.8(0.6)	85.3(1.7)	<.0001
Occupation, n (%)	79.1(0.8)	67.8(2.2)	<.0001	52.7(0.8)	40.6(2.3)	<.0001
Heath insurance			0.6182			0.0005
National health insurance (private)	38.1(1.1)	36.5(2.2)		35.2(0.8)	37.8(2.3)	
National health insurance (corporation)	60(1.1)	61.1(2.3)		62(0.8)	56.7(2.4)	
Medical aid	1.9(0.3)	2.4(0.7)		2.7(0.3)	5.4(0.9)	
National Basic Livelihood Security System			0.0016			<.0001
Current	1.9(0.3)	2.7(0.7)		2.8(0.3)	5.4(0.9)	
Previous	2.6(0.3)	5.6(1.2)		3(0.3)	6.1(1.1)	
Non-participating	95.5(0.4)	91.7(1.4)		94.2(0.4)	88.5(1.3)	

Abbreviations: BMI, body mass index; WC, weight circumference

Values are presented as mean (SEM) or percentage (SE)

Table 2 presents the distribution of blepharoptosis according to SES and occupation among men and women. The levels of household income and education were lower in both male and female subjects with blepharoptosis. The groups composed of skilled agricultural, forestry, and fishery workers; equipment and machine operators and craft and assembly workers; and elementary workers had the highest proportions of individuals with blepharoptosis, irrespective of gender (39.9% of men and 23.2% of women).

**Table 2:** Distribution of the prevalence of blepharoptosis according to socioeconomic status and occupation among men and women.

Socioeconomic status	Men			Women		
	Blepharoptosis			Blepharoptosis		
	No	Yes	P-value	No	Yes	P-value
**Household income level (%)**			<.0001			<.0001
Low	12.9(0.6)	22.7(1.7)		15.8(0.6)	43.2(2.1)	
Medium-low	26.4(0.8)	28.6(2.2)		27.5(0.8)	28.1(1.8)	
Medium-high	31.1(0.8)	25.3(1.8)		28.8(0.7)	17.5(1.6)	
High	29.6(0.9)	23.4(2)		27.9(0.8)	11.2(1.2)	
**Education level (%)**			<.0001			<.0001
≤ Elementary school	10.1(0.5)	29.3(2.2)		21.2(0.7)	69.1(2.4)	
Middle school	9.7(0.5)	15.1(1.6)		10.1(0.4)	10.4(1.1)	
High school	42.9(0.8)	30.7(2.2)		38.1(0.8)	14.3(1.5)	
≥ University	37.3(0.9)	24.9(2)		30.7(0.8)	6.3(1.5)	
**Occupation**			<.0001			<.0001
Manager, professional, and administrators; Clerks	29.3(0.8)	18.9(1.8)		19.9(0.6)	5.5(1.5)	
Service and sales workers	13.5(0.6)	8.9(1.2)		15.3(0.5)	9.8(1.2)	
Skilled agricultural, forestry, and fishery workers; Craft, equipment, machine operating, and assembling workers; Elementary workers	36.4(1)	39.9(2.3)		16.8(0.7)	23.2(1.6)	
Unemployed	20.8(0.7)	32.4(1.9)		48(0.7)	61.5(2.2)	

**Fig 1:**
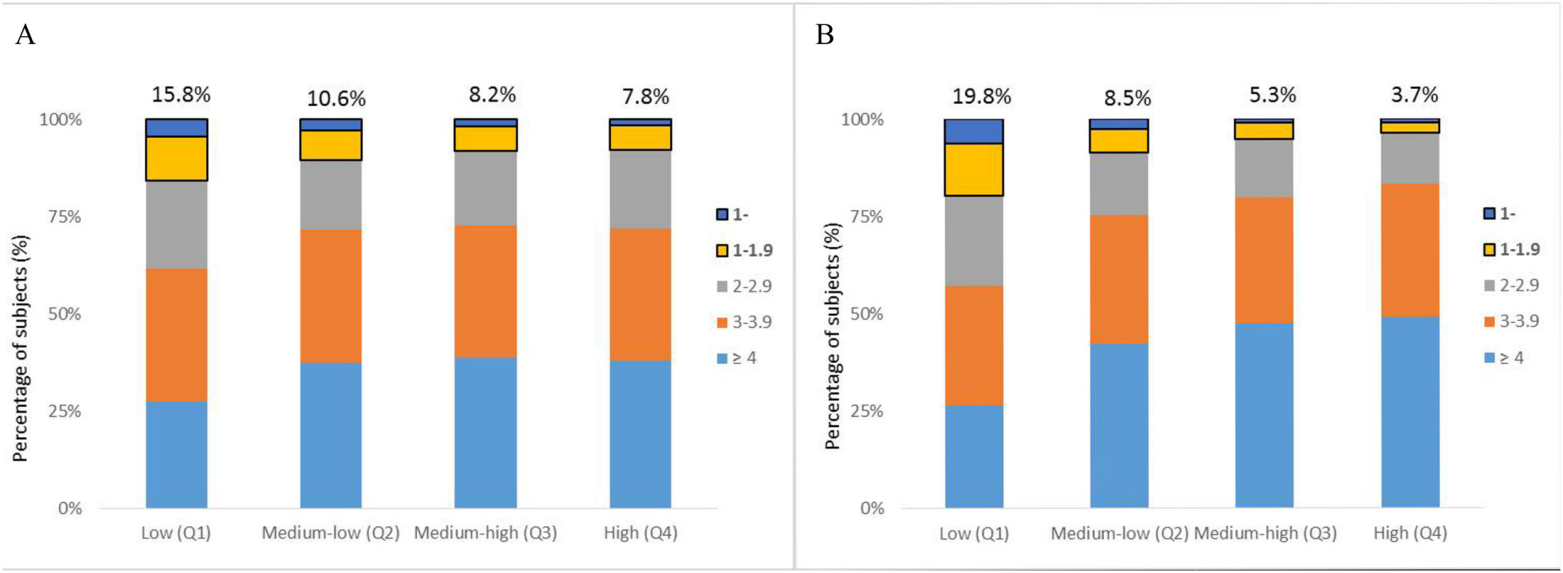
The prevalence of blepharoptosis by MRD 1 measurement according to level of household income. Household income was divided into quartiles and defined as low (Q1), medium-low (Q2), medium-high (Q3), and high (Q4) in men (A) and women (B). MRD, marginal reflex distance. *P*-value for trend < 0.001.

**Fig 2:**
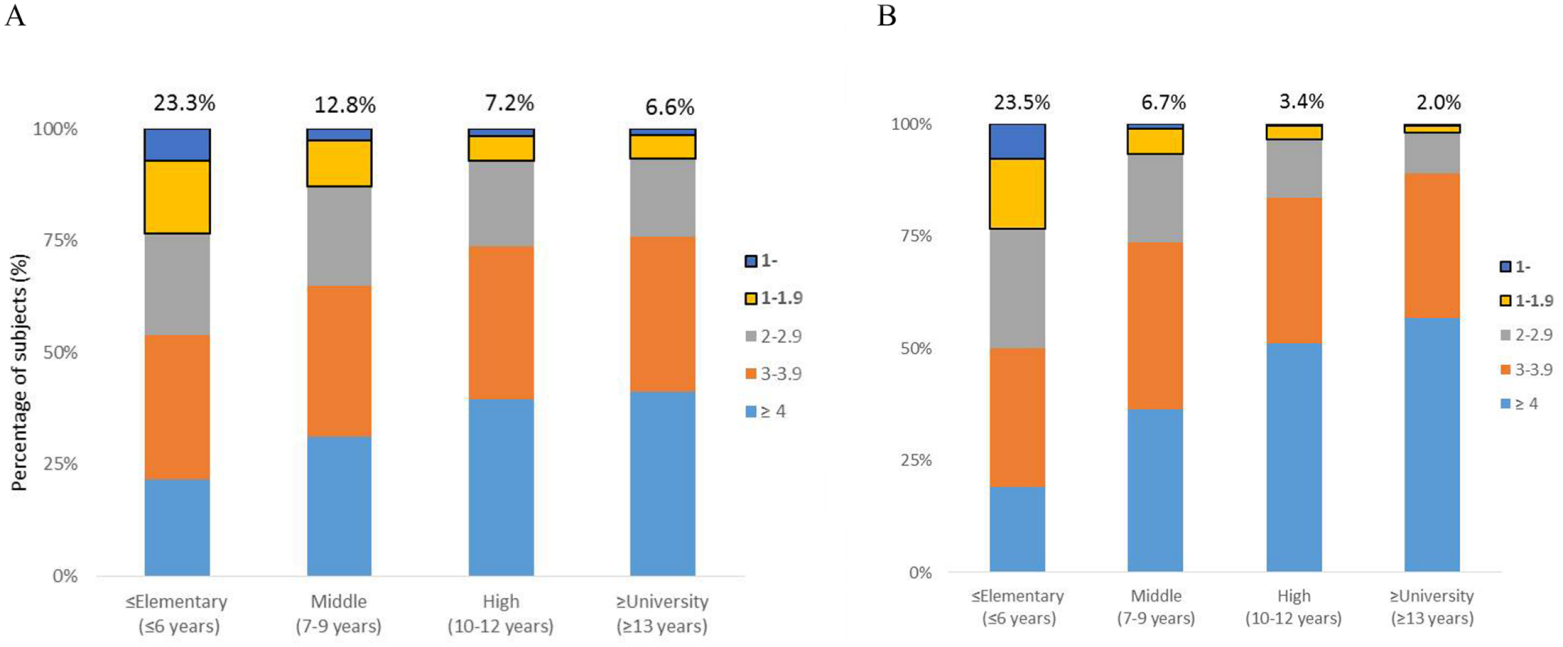
The prevalence of blepharoptosis by MRD 1 measurement according to education level. Education levels were divided into elementary school (≤ 6 years), middle school (7–9 years), high school (10–12 years), and university (≥ 13 years) in men (A) and women (B). MRD, marginal reflex distance. *P*-value for trend < 0.001.

Next, we analyzed the distribution of blepharoptosis according to household income and educational level in men and women. The proportion of subjects with blepharoptosis decreased significantly, from 15.8% and 19.8% in the lowest household income groups to 7.8% and 3.7% in the highest household income groups in men and women, respectively (Fig 1A and 1B). In addition, the rates of blepharoptosis decreased significantly as a level of education: the rates decreased from 23.3% of men and 23.5% of women with less than 6 years of education to 6.6% of men and 2.0% of women with more than 13 years of education (Fig 2A and 2B).

Table 3 shows the adjusted ORs (aORs) for the prevalence of blepharoptosis according to household income and educational level in men and women. In women, the aORs (95% CI) of blepharoptosis for the lowest vs. the highest quartiles of household income were 1.913 (1.407, 2.597) in Model 1, 1.884 (1.381, 2.569) in Model 2, and 1.894 (1.336, 2.685) in Model 3. This reflected a trend toward an association between lower household income and a higher prevalence of blepharoptosis in women. All adjusted analyses revealed significant associations between educational level and the prevalence of blepharoptosis in men and women. The aORs of blepharoptosis of men and women in the lowest vs. the highest quartiles were 1.648 (1.21, 2.244) and 2.062 (1.211, 3.512), respectively, in Model 1; 1.679 (1.227, 2.297) and 1.975 (1.145, 3.407), respectively, in Model 2; and 1.572 (1.113, 2.219) and 1.973 (1.153, 3.376), respectively, in Model 3.

**Table 3:** Odds ratios (95% confidence intervals) of blepharoptosis by socioeconomic status among men and women.

Socioeconomic status	Model 1^a^		Model 2^b^		Model 3^c^	
	Men	Women	Men	Women	Men	Women
Household income level						
Low	1.09(0.817,1.455)	1.913(1.409,2.597)	1.039(0.776,1.389)	1.884(1.381,2.569)	1.065(0.746,1.521)	1.894(1.336,2.685)
Medium-low	1.194(0.892,1.596)	2.026(1.492,2.751)	1.198(0.898,1.599)	1.986(1.459,2.704)	1.254(0.914,1.721)	1.825(1.28,2.602)
Medium-high	1.074(0.821,1.405)	1.585(1.169,2.148)	1.082(0.83,1.411)	1.557(1.144,2.118)	1.132(0.834,1.537)	1.464(1.039,2.063)
High	1	1	1	1	1	1
*P*-value for trend	0.3622	<.0001	0.4969	<.0001	0.4577	0.0002
Education level						
≤ Elementary school	1.648(1.21,2.244)	2.062(1.211,3.512)	1.679(1.227,2.297)	1.975(1.145,3.407)	1.572(1.113,2.219)	1.973(1.153,3.376)
Middle school	1.022(0.795,1.316)	1.238(0.768,1.996)	1.005(0.778,1.298)	1.206(0.75,1.94)	0.957(0.715,1.28)	1.185(0.723,1.941)
High school	1.279(0.897,1.824)	1.498(0.867,2.586)	1.27(0.886,1.819)	1.423(0.818,2.476)	1.266(0.85,1.886)	1.561(0.9,2.709)
University	1	1	1	1	1	1
*P*-value for trend	0.0015	0.0009	0.0012	0.002	0.0068	0.0012

^a^ Model 1 was adjusted for age.

^b^ Model 2 was adjusted for body mass index, smoking, and alcohol consumption.

^c^ Model 3 was additionally adjusted for diabetes, family history of eye disease, and energy, and fat intake.

**Fig 3:**
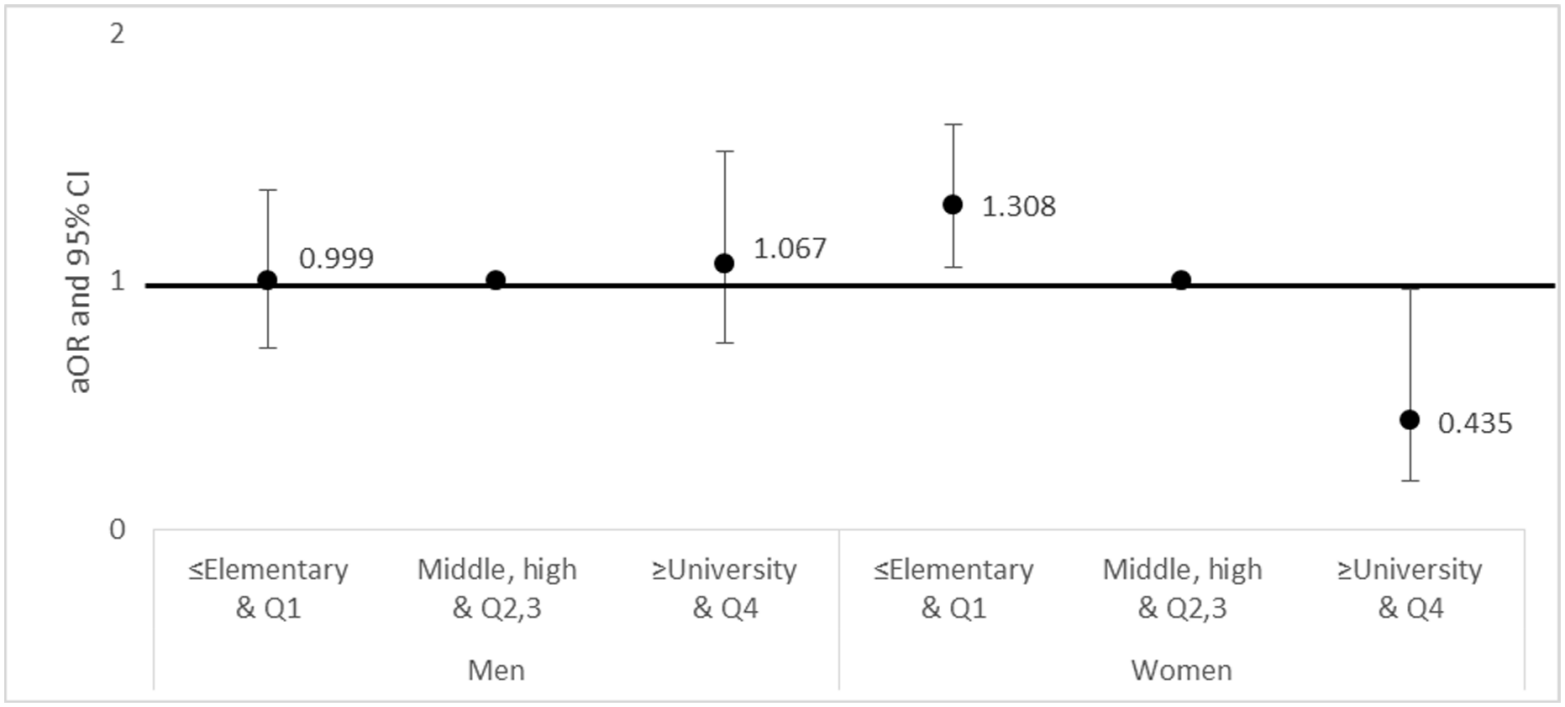
Adjusted OR and 95% CI for blepharoptosis by household income and education. Household income was divided into quartiles and defined as low (Q1), medium-low (Q2), medium-high (Q3), and high (Q4). Education level was classified as elementary school (≤ 6 years), middle school (7–9 years), high school (10–12 years), and university (≥ 13 years). The middle-income and middle-education groups served as reference groups. (A) Men (B) Women. OR, Odds ratio; CI, confidence interval. P < 0.0.

The aORs and 95% CIs for the prevalence of blepharoptosis by household income and education level are shown in Fig 3 according to gender. Among women, the low-income and low-education group had a higher prevalence of blepharoptosis (aOR 1.308, 95% CI 1.051–1.627) than the middle-income and middle-education group (reference group), whereas the high-income and high-education group showed a lower prevalence of blepharoptosis (aOR 0.435, 95% CI 0.196–0.966) than the reference group.

## Discussion

This study found a possible association between the prevalence of blepharoptosis and SES according to gender.

There has been a lack of global data regarding the association between the prevalence of blepharoptosis and SES. In a Nigerian study, the prevalence of blepharoptosis was highest among civil servants (38.5%), followed by students (26.9%) according to socioeconomic status during 2000–2006 [7].

In this study, low household income and low educational level were significantly associated with a high prevalence of blepharoptosis in women, whereas only educational level was significantly associated with blepharoptosis in men after adjusting for SES-related demographic variables (Table 3). As shown in Fig 3, after adjusting for education and household income, the negative association between SES and blepharoptosis was found only in women.

There are several possible explanations for the negative association between SES and blepharoptosis. Conditions that involve metabolic derangement, such as dyslipidemia, obesity, and diabetes have been shown to be possible risk factors for blepharoptosis [1, 2, 8, 9]. According to Shirado M. [9], dyslipidemia should be considered as a possible determinant of age-related ptosis. Atherogenic dyslipidemia may lead to a circulation disorder associated with the development of age-related blepharoptosis. Excessive weight is also a risk factor for blepharoptosis [1, 3, 4]. Mechanical ptosis develops when the eyelid is too heavy for the levator muscles to lift [1]. Diabetes is included in the risk factors for developing blepharoptosis [1, 3, 4]. Moon and Lee [8] investigated diabetes as an independent risk factor for blepharoptosis in the general Korean population, noting that metabolic dysregulation from diabetes may lead to malfunction or damage of the oculomotor or sympathetic nerves or to central nervous system abnormality [8]. Consistent with the aforementioned research, this study also showed that subjects with blepharoptosis were more likely to have diabetes and metabolic syndrome than subjects without blepharoptosis.

The associations between these metabolic dysregulation diseases and SES have been recently demonstrated [10, 11]. Indeed, a study investigating the relationship between SES and dyslipidemia among the Korean population found that lower SES and dyslipidemia were associated [11]. Kim et al. [6] found that the prevalence of diabetes was related to low SES, particularly in the young and middle-aged population in Korea. Chao at el. [10] reported that low SES was associated with the risk of central obesity. In addition, a cross-sectional study of SES and metabolic syndrome in Korean adults found that lower SES was associated with a higher risk of metabolic syndrome in women [12].

It is possible that household income and educational level, which comprised SES in this study, affect the prevalence of blepharoptosis for several reasons. Those with a low income may not be able to afford to participate in the activities that can affect the risk factors related to the prevalence of blepharoptosis [13, 14]. Education level also affects the acquisition and understanding of health knowledge. Therefore, people with a high education level have easier access to information and resources associated with health improvement, which contributes to reducing the risk factors for blepharoptosis [15–17].

In this study, SES influenced the prevalence of blepharoptosis differently according to gender. This gender specific relationship between SES and the prevalence of blepharoptosis was consistent with the association between SES and metabolic disease, in that women with lower SES were more likely to have blepharoptosis than women with high SES [11, 12]. This gender disparity developed due to the differential effects of household income and education level on men and women. Women with a high SES are more likely to engage in activities such as eating appropriate foods, exercising regularly, and weight control for health improvement because, in general, women have a higher level of interest in health issues than men [15, 17–19].

Injury is one factor related to traumatic blepharoptosis [1]. In Nigeria, trauma was the most common etiologic contributor to blepharoptosis [7]. In the study based on the fourth KNHANES [20], a negative correlation was found between the total injury experience rate and SES. Socioeconomic variables are affected by geographic factors, such as the degree to which there is a local infrastructure that promotes public safety (e.g., safe roads and recreational areas) [20]. Education level also affects safety consciousness, which helps people protect themselves from trauma [15–17]. Type of occupation is known to be a major contributor to SES [21]. In this study, men and women with blepharoptosis were significantly more likely to be engaged in jobs involving manual labor than were those without blepharoptosis. Manual labor is most likely related to a higher prevalence of blepharoptosis because manual labor carries a higher risk of injury [22–25].

This study has several limitations. First, we could not evaluate a causal relationship between SES and blepharoptosis because of the study’s cross-sectional and retrospective design. Second, there are no accurate diagnostic criteria for blepharoptosis in specifically Asian or Korean populations because facial anatomy differs depending on race [2]. Third, the levels of household income and education utilized to represent SES were insufficient to evaluate SES. Despite these limitations, this is the first epidemiologic study to examine the association between SES and blepharoptosis in the Korean population using nationally representative data.

## Conclusions

Socioeconomic disparities in the prevalence of blepharoptosis were found among Korean women. Given the multidimensionality of SES and the multiplicity of variables related to blepharoptosis, SES may emerge from future prospective studies as a predictor of this condition in women.
